# The SNP (rs2230500) in *PRKCH* Decreases the Risk of Carotid Intima-Media Thickness in a Chinese Young Adult Population

**DOI:** 10.1371/journal.pone.0040606

**Published:** 2012-07-11

**Authors:** Lijun Wu, Bo Xi, Dongqing Hou, Xiaoyuan Zhao, Junting Liu, Hong Cheng, Xin Zhou, Yue Shen, Xingyu Wang, Jie Mi

**Affiliations:** 1 Department of Epidemiology, Capital Institute of Pediatrics, Beijing, China; 2 Department of Maternal and Child Health Care, School of Public Health, Shandong University, Jinan, China; 3 Laboratory of Human Genetics, Beijing Hypertension League Institute, Beijing, China; University of Amsterdam Academic Medical Center, Netherlands

## Abstract

**Background:**

The SNP (rs2230500) in *PRKCH* (the gene encoding protein kinase C η) is associated with ischemic stroke and cerebral hemorrhage in the Chinese population, but the molecular mechanisms are not clear. The aim of the present study is to investigate the association between the SNP and atherosclerosis that is common pathological basis of ischemic stroke and cerebral hemorrhage.

**Methodology/Principal Findings:**

We examined the associations of the SNP with carotid intima-media thickness (CIMT), atherosclerosis diagnosed by CIMT, and factors related with inflammation in the Beijing Child Blood Pressure Study. A total of 1190 subjects participated in the follow-up study. The SNP was genotyped by allele-specific real-time PCR assay. The SNP (rs2230500) in *PRKCH* was significantly associated with CIMT (in far wall of left common carotid arteries, *P* = 0.016; in far wall of right common carotid arteries, *P* = 0.012) under a recessive model after adjustment for age, gender, smoking, and hypertension. The SNP was also significantly associated with complement C3 (*P* = 0.012) under a dominant model after adjustment for age, gender, and high sensitivity C-reactive protein.

**Conclusions/Significance:**

Our data provide evidence that the SNP (rs2230500) in *PRKCH* decreases the risk of CIMT that is a worthwhile predictor of stroke and complement system possibly mediates this process.

## Introduction

Cerebrovascular disease is the second leading cause of death worldwide and is threat to global public health [1], [2]. Stroke is also the dominant type of cardiovascular disease in China [3]. Studies have shown that atherosclerosis is the major cause of coronary heart diseases and cerebrovascular diseases including stroke [4]. Although atherosclerotic diseases such as stroke occur in the middle age and later, evidences have indicated that the pathophysiological process of atherosclerosis begins in childhood [5]–[7]. Therefore, identification and prevention of atherosclerosis in children and adolescents would be beneficial to reduce the risk of stroke.

Carotid intima-media thickness (CIMT), determined by B-mode ultrasound imaging, is a noninvasive measure of atherosclerosis [8], [9], and is a worthwhile predictor of future coronary heart disease and stroke independently of conventional vascular risk factors [10]–[12]. Some studies suggest that the prediction of CIMT may be better for stroke than for coronary heart disease [10], [13], [14].

It is well established that atherosclerosis is an inflammatory disease [15], [16], and the complement system are participated in the pathogenesis of atherosclerosis [17], [18]. Complement C3, one of the major plasma proteins of complement system, is increased in chronic inflammation and could be considered as an independent predictor of the progression of atherosclerotic diseases [19].

Protein kinase C η (PKC η) is a serine-threonine kinase and involved in the development and progression of atherosclerosis [20]. Serizawa M et al [21] and Kubo M et al [20] reported that the SNP (rs2230500) in *PRKCH* (the gene of PKC η) is associated with the risk of ischemic stroke in the Japanese population. The associations between this SNP and different types of stroke (ischemic stroke and cerebral hemorrhage) were found in the Chinese population [22], whether those associations were mediated through atherosclerosis is not clear.

We have previously conducted a prospective study of blood pressure in Chinese children and adolescents: the Beijing Child Blood Pressure Study (BBS), in 1987. This study is an on-going, population-based, and follow-up study of blood pressure. We investigated the association between the SNP (rs2230500) in *PRKCH* and CIMT, also potential associations between this SNP and factors related with inflammation in the cohort. The present study attempts to provide an analysis of epidemiological and genetic data towards the possible mechanism of the role of PKC η in atherosclerosis.

## Methods

### Ethics Statement

Our study was approved by the ethics committees of Capital Institute of Pediatrics (It is the full name of the ethics committees review board). We obtained informed written consent from parents or guardians on the behalf of the minors/children participants involved in our study when the baseline and follow-up study was conducted. We obtained informed written consent from all participants who are all adults when the latest follow-up study was conducted.

### Study Population

The BBS is an on-going, population-based, prospective, and follow-up study of blood pressure in Beijing children and young adults. The baseline study in 1987 included 5916 children and adolescents, aged 3–18 years. Baseline data included weight, height, heart rate, blood pressure, and left subscapularis skinfold. In 2005, 412 adult-participants of the study underwent clinical examinations, which included electrocardiogram, echocardiography, fundus examination, and biochemical detection. The latest follow-up study was conducted from 2010 to 2011, with 1190 subjects attending anthropometric measurement and medical examination. Participants were asked to complete questionnaires that included questions about smoking habits, drinking habits, whether suffer from coronary heart disease or stroke, and physical activities. Also, venipuncture blood samples were collected. We obtained written informed consent from all participants.

### Measurement of Anthropometric Parameters and Biochemical Analyses

Anthropometric measurement included weight, height, waist circumference, and fat mass percentage by a body composition analyzer (TBF-300A; TANITA). All instruments were validated following the standard methods of the manufacturers [23]. BMI was calculated as weight in kilograms divided by the square of height in meters. Total cholesterol, triglycerides, high-density lipoprotein cholesterol (HDL), low-density lipoprotein cholesterol (LDL), complement C3, high sensitivity C-reactive protein (hs-CRP), and glycated haemoglobin (HbA1C), were analyzed by an automatic biochemical analyzer (Hitachi 7020) using a kit assay (SEKISUI medical technology Ltd., Tokyo, Japan).

### Measurement of Blood Pressure

Blood pressure was measured by auscultation using a standard clinical sphygmomanometer. Measurements were taken on the right arm in a sitting position with the elbow at the level of the right atrium, using an appropriately sized cuff. Systolic blood pressure (SBP) was determined by the onset of the “tapping” Korotkoff sounds (K1) and diastolic blood pressure (DBP) was determined by the fourth Korotkoff sound (K4). Three consecutive measurements were performed and the mean of the three readings was used for analysis. Hypertension was defined as participants having current or past anti-hypertensive medication, SBP≥140 mm Hg, or DBP≥90 mm Hg.

### Carotid Artery Ultrasound Examinations

B-mode ultrasound studies of left and right carotid artery were performed by using a portable ultrasound machine (SonoSite M-Turbo, SonoSite, Inc. USA) with a 13-MHz linear-array transducer in a stable field center (Beijing Children Center for Chronic Disease Prevention and Management). The fixed parameters of the portable ultrasound machine were used in the study, and the machine was debugged before detecting B-mode ultrasound. Two ultrasonographers were trained centrally by a certified ultrasonographer and accomplished the inter-observers variance study (IVOS) in pilot study. The study was conducted from 2010 to 2011. Longitudinal images of the common carotid artery (CCA) were acquired according to a standardized protocol. The optimized images of left and right carotid artery intima-media thickness (IMT) were selected and frozen at the end of diastolic. In 1 to 1.5 cm approximately proximal to the bifurcation, the images were magnified by a solution box function (zoom). IMT of CCA was measured with automated ultrasonic software–Calcs (to trace the lumen-intima and media-adventitia interfaces) in near and far wall respectively on both the left and right sides in an area free of atherosclerotic plaque. The value of IMT of CCA in far wall of each side was used for analysis. The near and far walls of CCA, carotid bifurcations and origins of the external and internal carotids arteries in both sides were scanned both longitudinally and transversely for the presence of atherosclerotic plaque, defined by the appearance of largest focal lesion (protrusion into the lumen of ≥1.5 mm thickness), shape and texture in longitudinal images. Then plaques were required to confirm in cross-sectional views of the lumen. The largest extent of plaque was measured with ultrasonic calipers and recorded in left or right side. According to acoustic shadowing of B-mode ultrasound, plaques that appeared echogenic, echolucent, or both echogenic and echolucent, were classified as calcified plaques, soft plaques, or mixed plaques, respectively [24]. In IVOS of our center, Pearson correlation coefficients based on 50 repeated studies on IMT in far wall of CCA were 0.900 for the left and 0.917 for the right, respectively. Assessment of early atherosclerosis was based on the value of the IMT in far wall of left or right CCA. CIMT category [25] can be found in Table S1.

### Genotyping

Genomic DNA was extracted from peripheral blood white cells using QIAamp® DNA Blood Mini Kit, according to the instructions of the manufacturer (QIAGEN Inc., Valencia, CA). The SNP (rs2230500) in *PRKCH* was genotyped by allele-specific real-time polymerase chain reaction (PCR) [26] using Gene-Amp 5700 Sequence Detector (Applied Biosystem). The primers and procedure of PCR amplifications can be found in Method S1. The genotyping call rate was 98%. For validating the accuracy of genotyping, we sent 30 samples to direct sequencing and observed 100% concordance between two genotyping methods.

### Statistical Analysis

Continuous variables were presented as mean±SD, and categorical variables were presented as percentages. Hardy–Weinberg equilibrium was assessed by the Chi-square test. ANCOVA were used to calculate mean differences in CIMT according to genotypes with adjustment for age, gender, smoking, and hypertension. Adjusted odds ratios (ORs) for atherosclerosis diagnosed by CIMT were performed by logistic regression with genotypes, age, gender, smoking, and hypertension as the independent variables. ANCOVA were also used to calculate mean differences in complementC3 according to genotypes with age and gender were used as covariate. Data were analyzed using SPSS statistical software (version 13.0, SPSS Inc., Chicago, Illinois). *P*<0.05 was used to indicate statistically significant difference. Power calculation was performed using Quanto software (http://hydra.usc.edu/gxe/).

## Results

### Participant Characteristics

A total of 1190 participants were recruited in the latest follow-up study and had DNA samples available. The characteristics of the study participants are summarized in Table 1. The participants of atherosclerosis diagnosed by IMT in far wall of left CCA (L_CIMT) or right CCA (R_CIMT) were 264 and 226, respectively. Age-specific CIMT cutoffs [25] can be found in Table S1.

**Table 1:** Basic characteristics of participants in this study.

	All
No. of subjects	1190
Male, %	56.5
Age, y	34.4 (3.6)
Systolic blood pressure, mm Hg	114.5 (15.7)
Diastolic blood pressure, mm Hg	72.8 (12.3)
Hypertension, %	12.2
Body mass index, kg/m^2^	24.7 (4.2)
L_CIMT, mm	0.542 (0.069)
R_CIMT, mm	0.531 (0.067)
Atherosclerosis diagnosed by L_CIMT, %	22.2
Atherosclerosis diagnosed by R_CIMT, %	19.0
Carotid plaque group, %	3.1
Complement C3, mg/dL	136.6 (31.9)
High sensitivity C-reactive protein, mg/dL	1.3 (0.6–2.4)
Total cholesterol, mmol/L	4.7 (1.0)
Triglycerides, mmol/L	1.2 (0.8–1.9)
High-density lipoprotein cholesterol, mmol/L	1.3 (0.3)
Low-density lipoprotein cholesterol, mmol/L	2.8 (0.7)
HbA1C, %	5.9 (0.8)
Smokers, %	35.5

L_CIMT indicates IMT in far wall of left common carotid arteries; R_CIMT, IMT in far wall of right common carotid arteries.

Values are means (SDs), geometric means (quartile deviations), or percentages of subjects, as appropriate.

### Associations of the SNP with CIMT and Atherosclerosis

We genotyped the SNP (rs2230500) in *PRKCH* in the cohort and the SNP was tested to be in Hardy–Weinberg equilibrium (*P* = 0.97). Associations of the SNP with CIMT and atherosclerosis are shown in Table 2 and 3. There was a significant difference between two sides of CIMT. Therefore, the statistical analyses based on the value of each side of CIMT were performed. Table 2 shows that the mean of CIMT with AA genotype is the lowest in the three genotypes. As smoking and hypertension are traditional risk factors of CIMT, we also adjusted these factors besides age and gender. The SNP (rs2230500) in *PRKCH* was significantly associated with L_CIMT (*P* = 0.016) and R_CIMT (*P* = 0.012) under a recessive model after adjustment for age, gender, smoking, and hypertension.

**Table 2:** Associations of rs2230500 with CIMT.

Genetic model	L_CIMT							R_CIMT						
	CIMT*, mm	*P* *	R^2^ †	R^2^ *	CIMT‡, mm	*P* ‡	R^2^ ‡	CIMT*, mm	*P* *	R^2^ †	R^2^ *	CIMT‡, mm	*P* ‡	R^2^ ‡
Genotypic model														
GG(n = 755)	0.543 (0.055)				0.545 (0.055)			0.533 (0.055)				0.533 (0.055)		
AG(n = 386)	0.542 (0.059)	0.792			0.543 (0.059)	0.723		0.530 (0.059)	0.335			0.529 (0.059)	0.388	
AA(n = 49)	0.527 (0.063)	0.068	0.007	0.191	0.521 (0.063)	0.015	0.189	0.515 (0.063)	0.046	0.007	0.193	0.508 (0.063)	0.0088	0.181
Global *P* value		0.188				0.052			0.107				0.028	
Dominant model														
GG	0.543 (0.055)				0.545 (0.055)			0.533 (0.055)				0.533 (0.055)		
AG+AA	0.540 (0.101)	0.452	0.001	0.189	0.541 (0.101)	0.315	0.185	0.528 (0.101)	0.149	0.002	0.191	0.527 (0.101)	0.127	0.177
Recessive model														
AG +GG	0.543 (0.042)				0.544 (0.042)			0.532 (0.042)				0.532 (0.042)		
AA	0.527 (0.063)	0.071	0.007	0.191	0.521 (0.063)	0.016	0.189	0.515 (0.063)	0.060	0.007	0.192	0.508 (0.063)	0.012	0.180

L_CIMT indicates IMT in far wall of left common carotid arteries; R_CIMT, IMT in far wall of right common carotid arteries.

* Adjusted for age, gender, and hypertension diagnosed only on SBP and/or DBP.

† The variation explained by rs2230500.

‡ Adjusted for age, gender, smoking, and hypertension diagnosed only on SBP and/or DBP.

CIMT are expressed as mean (SD).

**Table 3:** Associations of rs2230500 with atherosclerosis diagnosed by CIMT.

Genetic model	Atherosclerosis diagnosed by L_CIMT							Atherosclerosis diagnosed by R_CIMT						
	OR (95% CI)*	*P* *	R^2^ †	R^2^ *	OR (95% CI)‡	*P* ‡	R^2^ ‡	OR (95% CI)*	*P* *	R^2^ †	R^2^ *	OR (95% CI)‡	*P* ‡	R^2^ ‡
Genotypic model														
GG(n = 755)	1				1			1				1		
AG(n = 386)	1.12 (0.82–1.52)	0.479			1.21 (0.88–1.66)	0.243		1.01 (0.73–1.41)	0.941			1.07 (0.76–1.51)	0.709	
AA(n = 49)	0.62 (0.27–1.43)	0.257	0.003	0.104	0.47 (0.18–1.23)	0.124	0.099	0.60 (0.24–1.48)	0.267	0.003	0.122	0.44 (0.15–1.27)	0.127	0.112
Dominant model														
GG	1				1			1				1		
AG+AA	1.05 (0.78–1.42)	0.734	0.0001	0.102	1.11 (0.82–1.51)	0.509	0.093	0.96 (0.70–1.32)	0.805	0.001	0.121	0.99 (0.71–1.38)	0.931	0.107
Recessive model														
AG +GG	1				1			1				1		
AA	0.59 (0.26–1.37)	0.220	0.003	0.103	0.44 (0.17–1.15)	0.093	0.097	0.60 (0.25–1.46)	0.260	0.002	0.122	0.43 (0.15–1.23)	0.115	0.112

L_CIMT indicates IMT in far wall of left common carotid arteries; R_CIMT, IMT in far wall of right common carotid arteries.

* Adjusted for age, gender, and hypertension diagnosed only on SBP and/or DBP.

† The variation explained by rs2230500.

‡ Adjusted for age, gender, smoking, and hypertension diagnosed only on SBP and/or DBP.

ORs for atherosclerosis diagnosed by L_CIMT (OR = 0.44, 95% confidence interval [95% CI], 0.17 to 1.15; *P* = 0.093) and R_CIMT (OR = 0.43, 95% CI, 0.15 to 1.23; *P* = 0.115) did not reach statistical significance. With the assumed effect size of 0.44 and 0.43, respectively, for an allele frequency of 0.203, the powers for the SNP were 0.464 and 0.435. This could be attributable to the small sample size (participants of atherosclerosis diagnosed by L_CIMT or R_CIMT were 264 and 226, respectively) in our study. Studies with greater sample size are needed to examine the associations.

### Association of the SNP with Complement C3

Table 4 shows the association of rs2230500 with complement C3. The SNP was significantly associated with complement C3 (*P* = 0.012) under a dominant model after age and gender adjustment. The results showed that the A allele of SNP decreased the concentration of complement C3 (Table 4). Considering the concentration of complement C3 is linearly associated with serum hs-CRP [27], we also adjusted for hs-CRP, and the significant association remained (*P* = 0.012).

**Table 4:** Association of rs2230500 with complement C3.

Genetic model	n = 1190						
	ComplementC3(mg/dL)*	*P* *	R^2^ †	R^2^ *	ComplementC3(mg/dL)‡	*P* ‡	R^2^ ‡
Genotypic model							
GG(n = 755)	138.6 (35.7)				138.5 (33.0)		
AG(n = 386)	133.8 (35.4)	0.028			133.8 (33.4)	0.024	
AA(n = 49)	129.4 (34.3)	0.071	0.008	0.026	130.8 (32.9)	0.115	0.086
Global *P* value		0.029				0.036	
Dominant model							
GG	138.6 (35.7)				138.5 (33.0)		
AG+AA	133.3 (33.4)	0.012	0.007	0.026	133.4 (20.8)	0.012	0.085
Recessive model							
AG +GG	136.9 (33.8)				136.9 (33.8)		
AA	129.4 (34.3)	0.133	0.003	0.021	130.8 (32.9)	0.207	0.081

* Adjusted for age and gender.

† The variation explained by rs2230500.

‡ Adjusted for age, gender, and hs-CRP.

ComplementC3 are expressed as mean (SD).

### Associations of the SNP with Blood Lipids, Blood Pressure, BMI, and hs-CRP

We analyzed the associations of the SNP with clinical parameters including blood lipids (total cholesterol, HDL, LDL, and triglycerides), blood pressure, BMI, and hs-CRP. There were no statistical significances between the SNP and these clinical parameters (data can be found in Table S2 and S3). Blood pressure and BMI were measured in both childhood and adulthood. The associations between the SNP and changes of hypertension and obesity were also analyzed. There were no statistical significances between the SNP and changes of these phenotypes (data can be found in Table S3). Studies with greater sample size are needed to confirm these associations.

### Influence of Hypertension on Associations between the SNP and Atherosclerosis

The effect of interaction between genotypes and hypertension status on the atherosclerosis diagnosed by CIMT was assessed by binary logistic regression model with genotypes, hypertension status, their interaction, age and gender as the independent variables under recessive model. The results can be found in Table S4. In normal blood pressure population, the risk of atherosclerosis with AA genotype was lower than that of GG+AG genotype. Compared with hypertensives with same genotype, the ORs of atherosclerosis diagnosed by L_CIMT or R_CIMT of normal blood pressure participants with GG+AG genotype were 0.43 (95% CI, 0.28 to 0.67; *P*<0.001) and 0.33 (95% CI, 0.21 to 0.52; *P*<0.001), respectively. It suggests that hypertension increases the genetic susceptibility.

## Discussion

In this study, we examined the SNP (rs2230500) in *PRKCH* in the Chinese young adult population. Our results indicated that this SNP is associated with CIMT under a recessive model, and there is a statistically significant association of rs2230500 with complement C3.

Atherosclerosis progression begins in children and adolescents, and atherosclerotic diseases occur later in life [5]–[7], so prevention of atherosclerosis earlier in life would be favorable to reduce the incidence and disease burden of coronary heart diseases and cerebrovascular diseases.

The complement system comprises approximately 30 components including C3 that is one of the major members of the complement system. Complement C3 is considered as a signal of the inflammatory process and future risk of developing cardiovascular diseases [17], [19], [28]. Concentrations of C3 is independently and linearly associated with serum CRP that activates the complement pathway [27], [29]. Therefore, we adjusted for hs-CRP in our study, and the significant association between the SNP (rs2230500) and complement C3 remained (*P* = 0.012). However, no significant association was found between the SNP and hs-CRP (data can be found in Table S2). Studies with greater sample size are needed to confirm the association, because the power was 0.122, assuming the effect size of −0.19 with the frequency of 0.203.

Protein kinase C are participated in a wide variety of signaling pathways, and regulates multiple important cellular functions, such as proliferation, differentiation, and apoptosis [20], [30]. PKC η, a member of the PKC family, is expressed mainly in vascular endothelial cells [20] and is involved in the induction of inducible nitric oxide synthase (iNOS) and nitric oxide (NO) release [31]. *PRKCH* encoding PKC η is located in chromosome 14q22-q23 in human. The nonsynonymous SNP rs2230500 that lies in exon 9 and within the ATP-binding site of PKC η increases the kinase activity [20].

Previous studies showed that the SNP (rs2230500) in *PRKCH* is associated with the risk of ischemic stroke in the Japanese population [20] and different types of stroke in the Chinese population [22]. However, whether those associations were mediated through atherosclerosis is not clear. In the current study, the association between the SNP (rs2230500) in *PRKCH* and complement C3 was found firstly, and the results showed that the SNP is associated with CIMT that is a subclinical indicator of atherosclerosis. Our results suggest that *PRKCH* is involved in atherosclerosis, whether the association is mediated through complement system is yet to be determined.

### Conclusions

We demonstrated for the first time that the SNP (rs2230500) in *PRKCH* is associated with CIMT used as a subclinical phenotype for atherosclerosis and complement C3 considered as a signal of the inflammatory process. These novel findings provided important evidence that PKC η is involved in atherosclerosis and complement system possibly mediates this process.

## Supporting Information

Table S1 **Atherosclerosis diagnosed by CIMT.**
(DOC)

Table S2 **Associations of rs2230500 with blood lipids, blood pressure, BMI, and hs-CRP.**
(DOC)

Table S3 **Associations of rs2230500 with dyslipidemias, hypertension, and obesity.**
(DOC)

Table S4 **The effect of interaction between genotypes and hypertension status on the atherosclerosis diagnosed by CIMT.**
(DOC)

Method S1 **Allele-specific real-time PCR assay.**
(DOC)

## References

[1] World Health Organization (2008). World Health Statistics 2008.. Geneva, Switzerland: World Health Organization.

[2] Bonow RO, Smaha LA, Smith SC, Mensah GA, Lenfant C (2002). World Heart Day 2002: the international burden of cardiovascular disease: responding to the emerging global epidemic.. Circulation.

[3] Reddy KS (2004). Cardiovascular disease in non-Western countries.. N Engl J Med.

[4] Scott J (2004). Pathophysiology and biochemistry of cardiovascular disease.. Curr Opin Genet Dev.

[5] Hixson JE (1991). Apolipoprotein E polymorphisms affect atherosclerosis in young males. Pathobiological Determinants of Atherosclerosis in Youth (PDAY) Research Group.. Arterioscler Thromb Vasc Biol.

[6] Urbina EM, Williams RV, Alpert BS, Collins RT, Daniels SR (2009). American Heart Association Atherosclerosis, Hypertension, and Obesity in Youth Committee of the Council on Cardiovascular Disease in the Young. Noninvasive assessment of subclinical atherosclerosis in children and adolescents: recommendations for standard assessment for clinical research: a scientific statement from the American Heart Association.. Hypertension.

[7] McGill HC, McMahan CA (1998). Determinants of atherosclerosis in the young: Pathobiological Determinants of Atherosclerosis in Youth (PDAY) Research Group.. Am J Cardiol.

[8] de Groot E, Hovingh GK, Wiegman A, Duriez P, Smit AJ (2004). Measurement of arterial wall thickness as a surrogate marker for atherosclerosis.. Circulation.

[9] Lorenz MW, von Kegler S, Steinmetz H, Markus HS, Sitzer M (2006). Carotid intima-media thickening indicates a higher vascular risk across a wide age range: prospective data from the Carotid Atherosclerosis Progression Study (CAPS).. Stroke.

[10] Simon A, Megnien JL, Chironi G (2010). The value of carotid intima-media thickness for predicting cardiovascular risk.. Arterioscler Thromb Vasc Biol.

[11] Kastelein JJ, de Groot E (2008). Ultrasound imaging techniques for the evaluation of cardiovascular therapies.. Eur Heart J.

[12] Polak JF, Pencina MJ, O’Leary DH, D’Agostino RB (2011). Common carotid artery intima-media thickness progression as a predictor of stroke in multi-ethnic study of atherosclerosis.. Stroke.

[13] Bots ML, Hoes AW, Koudstaal PJ, Hofman A, Grobbee DE (1997). Common carotid intima-media thickness and risk of stroke and myocardial infarction: the Rotterdam Study.. Circulation.

[14] O’Leary DH, Polak JF, Kronmal RA, Manolio TA, Burke GL (1999). Cardiovascular Health Study Collaborative Research Group. Carotid-artery intima and media thickness as a risk factor for myocardial infarction and stroke in older adults.. N Engl J Med.

[15] Hansson GK (2005). Inflammation, atherosclerosis, and coronary artery disease.. N Engl J Med.

[16] Ross R (1999). Atherosclerosis - an inflammatory disease.. N Engl J Med.

[17] Millonig G, Malcom GT, Wick G (2002). Early inflammatory-immunological lesions in juvenile atherosclerosis from the Pathobiological Determinants of Atherosclerosis in Youth (PDAY)-study.. Atherosclerosis.

[18] Oksjoki R, Kovanen PT, Pentikäinen MO (2003). Role of complement activation in atherosclerosis.. Curr Opin Lipidol.

[19] Szeplaki G, Prohaszka Z, Duba J, Rugonfalvi-Kiss S, Karadi I (2004). Association of high serum concentration of the third component of complement (C3) with pre-existing severe coronary artery disease and new vascular events in women.. Atherosclerosis.

[20] Kubo M, Hata J, Ninomiya T, Matsuda K, Yonemoto K (2007). A nonsynonymous SNP in *PRKCH* (protein kinase C eta) increases the risk of cerebral infarction.. Nat Genet.

[21] Serizawa M, Nabika T, Ochiai Y, Takahashi K, Yamaguchi S (2008). Association between *PRKCH* gene polymorphisms and subcortical silent brain infarction.. Atherosclerosis.

[22] Wu L, Shen Y, Liu X, Ma X, Xi B (2009). The 1425G/A SNP in *PRKCH* is associated with ischemic stroke and cerebral hemorrhage in a Chinese population.. Stroke.

[23] World Health Organization (1995). Physical status: the use and interpretation of anthropometry. Report of a WHO Expert Committee. Technical Report Series No. 854.. Geneva, Switzerland: World Health Organization.

[24] Zureik M, Bureau JM, Temmar M, Adamopoulos C, Courbon D (2003). Echogenic carotid plaques are associated with aortic arterial stiffness in subjects with subclinical carotid atherosclerosis.. Hypertension.

[25] Hu DY, Guo YF (2008). Non-invasive detection of arterial function evaluation of the clinical significance of China expert consensus. Cardiovascular disease prevention guidelines and consensus 2008.. People’s Health Publishing House.

[26] Germer S, Holland MJ, Higuchi R (2000). High-throughput SNP allele-frequency determination in pooled DNA samples by kinetic PCR.. Genome Res.

[27] Onat A, Can G, Rezvani R, Cianflone K (2011). Complement C3 and cleavage products in cardiometabolic risk.. Clin Chim Acta.

[28] Muscari A, Bozzoli C, Puddu GM, Sangiorgi Z, Dormi A (1995). Association of serum C3 levels with the risk of myocardial infarction.. *Am J Med*.

[29] Dernellis J, Panaretou M (2006). Effects of C-reactive protein and the third and fourth components of complement (C3 and C4) on incidence of atrial fibrillation.. Am J Cardiol.

[30] Nishizuka Y (1995). Protein kinase C and lipid signaling for sustained cellular responses.. FASEB J.

[31] Pham TN, Brown BL, Dobson PR, Richardson VJ (2003). Protein kinase C-eta (PKC-eta) is required for the development of inducible nitric oxide synthase (iNOS) positive phenotype in human monocytic cells.. Nitric Oxide.

