# Supplementary material for: The SNP (rs2230500) in PRKCH Decreases the Risk of Carotid Intima-Media Thickness in a Chinese Young Adult Population
Source: PLoS One. 2012 Jul 11;7(7):e40606. doi: 10.1371/journal.pone.0040606 (PMC3394745; doi:10.1371/journal.pone.0040606)
Supplement: Table S1 — Atherosclerosis diagnosed by CIMT. (DOC) [file pone.0040606.s001.doc]

**Supporting Information**

**Table S1. Atherosclerosis diagnosed by CIMT.**

| Age, y | CIMT, mm | AS_CIMT* |
| --- | --- | --- |
| 20-29 | <0.5 | No |
|  | >=0.5 | Yes |
| 30-39 | <0.6 | No |
|  | >=0.6 | Yes |
| 40-49 | <0.7 | No |
|  | >=0.7 | Yes |
| 50-59 | <0.8 | No |
|  | >=0.8 | Yes |
| >60 | <0.9 | No |
|  | >=0.9 | Yes |

*AS_CIMT indicates atherosclerosis diagnosed by CIMT.
