# Supplementary material for: The SNP (rs2230500) in PRKCH Decreases the Risk of Carotid Intima-Media Thickness in a Chinese Young Adult Population
Source: PLoS One. 2012 Jul 11;7(7):e40606. doi: 10.1371/journal.pone.0040606 (PMC3394745; doi:10.1371/journal.pone.0040606)
Supplement: Table S2 — Associations of rs2230500 with blood lipids, blood pressure, BMI, and hs-CRP. (DOC) [file pone.0040606.s002.doc]

**Supporting Information**

**Table S2. Associations of rs2230500 with blood lipids, blood pressure, BMI, and hs-CRP.**

| Factors | GG(n=755) | AG(n=386) | AA(n=49) | AG +GG (n=1141) | *P** | R^2^† | Adjusted R^2^ |
| --- | --- | --- | --- | --- | --- | --- | --- |
| Total cholesterol , mmol/L ‡ | 4.7 (1.1) | 4.7 (1.0) | 4.8 (0.9) | 4.7 (0.9) | 0.517 | 0.001 | 0.063 |
| HDL, mmol/L ‡ | 1.3 (0.4) | 1.3 (0.3) | 1.3 (0.3) | 1.3 (0.3) | 0.519 | 0.001 | 0.157 |
| LDL, mmol/L ‡ | 2.8 (0.8) | 2.8 (0.8) | 2.8 (0.7) | 2.8 (0.7) | 0.821 | 0.001 | 0.073 |
| Triglycerides, mmol/L ‡ | 1.3 (0.8-2.0) | 1.2 (0.7-1.8) | 1.3 (0.7-1.8) | 1.2 (0.8-1.9) | 0.83 | 0.001 | 0.209 |
| Adulthood SBP, mmHg § | 114.7 (14.8) | 114.3 (12.8) | 112.3 (12.0) | 114.5 (12.0) | 0.194 | 0.002 | 0.415 |
| Childhood SBP, mmHg § | 105.2 (11.8) | 105.2 (10.1) | 102.6 (2.5) | 105.2 (9.5) | 0.057 | 0.008 | 0.389 |
| Adulthood DBP, mmHg § | 72.7 (11.9) | 73.1 (10.3) | 71.8 (2.7) | 72.8 (9.7) | 0.466 | 0.002 | 0.377 |
| Childhood DBP k4, mmHg § | 66.4 (9.8) | 65.9 (8.4) | 66.0 (8.0) | 66.2 (8.0) | 0.797 | 0.002 | 0.212 |
| Adulthood BMI, kg/m^2^ ‡ | 24.7 (4.8) | 24.6 (4.1) | 24.9 (3.9) | 24.7 (3.9) | 0.756 | 0.001 | 0.141 |
| Childhood BMI, kg/m^2^ ‡ | 17.1 (3.1) | 17.1 (2.7) | 16.6 (2.5) | 17.1 (2.5) | 0.218 | 0.006 | 0.337 |
| hs-CRP, mg/dL ‡ | 1.2 (0.6-2.3) | 1.3 (0.6-2.6) | 1.2 (0.6-1.8) | 1.3 (0.6-2.4) | 0.748 | 0.001 | 0.036 |

*Under recessive model.

†The variation explained by rs2230500.

‡Adjusted for age and gender.

§Adjusted for age, gender, and BMI.

HDL indicates high density lipoprotein; LDL, low density lipoprotein; SBP, systolic blood pressure; DBP, diastolic blood pressure; BMI, body mass index; hs-CRP, high sensitivity C-reactive protein.

HDL, LDL, SBP, DBP, and BMI are means (SDs). Triglycerides and hs-CRP are geometric means (quartile deviations).
