# Supplementary material for: The SNP (rs2230500) in PRKCH Decreases the Risk of Carotid Intima-Media Thickness in a Chinese Young Adult Population
Source: PLoS One. 2012 Jul 11;7(7):e40606. doi: 10.1371/journal.pone.0040606 (PMC3394745; doi:10.1371/journal.pone.0040606)
Supplement: Table S3 — Associations of rs2230500 with dyslipidemias, hypertension, and obesity. (DOC) [file pone.0040606.s003.doc]

**Supporting Information**

**Table S3. Associations of rs2230500 with dyslipidemias, hypertension, and obesity.**

| Factors | OR (95% CI) | *P* | R^2^* | Adjusted R^2^ |
| --- | --- | --- | --- | --- |
| High total cholesterol † | 0.93 (0.22-4.01) | 0.923 | 0.0003 | 0.082 |
| Low HDL † | 1.54 (0.72-3.28) | 0.265 | 0.0003 | 0.132 |
| High LDL † | 0.52 (0.07-3.90) | 0.524 | 0.002 | 0.078 |
| High triglycerides † | 0.85(0.34-2.13) | 0.730 | 0.001 | 0.179 |
| Adulthood hypertension ‡ | 0.25 (0.05-1.26) | 0.093 | 0.008 | 0.533 |
| Childhood hypertension ‡ | 1.25 (0.37-4.20) | 0.722 | 0.001 | 0.053 |
| AHBP ‡ | 0.17 (0.02-1.74) | 0.135 | 0.009 | 0.528 |
| Adulthood obesity † | 1.84 (0.83-4.08) | 0.131 | 0.001 | 0.190 |
| Childhood obesity † | 0.49 (0.07-3.67) | 0.487 | 0.001 | 0.035 |
| AOB † | 2.20 (0.94-5.14) | 0.069 | 0.003 | 0.175 |

*The variation explained by rs2230500.

†Adjusted for age and gender, under recessive model.

‡Adjusted for age, gender, and BMI, under recessive model.

AHBP indicates adulthood hypertension and childhood with normal blood pressure; AOB, adulthood obesity and childhood with normal BMI.

Dyslipidemias were diagnosed by the Chinese blood lipids cutoffs (Joint Committee for Developing Chinese Guidelines on Prevention and Treatment of Dyslipidemia in Adults. (2007) Chinese guidelines on prevention and treatment of dyslipidemia in adults. Zhonghua Xin Xue Guan Bing Za Zhi 35:390–419). Childhood hypertension was diagnosed by the Chinese age- and sex-specific blood pressure cutoffs (Mi J, Wang TY, Meng LH, Zhu GJ, Han SM, et al. (2010) Development of blood pressure reference standards for Chinese children and adolescents. Chin J Evid Based Pediatr 5: 4–14). Childhood obesity was diagnosed by the Chinese age- and sex-specific BMI cutoffs (Ji CY; Working Group on Obesity in China. (2005) Report on childhood obesity in China (1)--body mass index reference for screening overweight and obesity in Chinese school-age children. Biomed Environ Sci. 18:390–400). Adulthood obesity was diagnosed by the Chinese BMI cutoffs (Zhou B; Coorperative Meta-Analysis Group Of China Obesity Task Force. (2002) Predictive values of body mass index and waist circumference to risk factors of related diseases in Chinese adult population. Zhonghua Liu Xing Bing Xue Za Zhi 23:5–10).
