# Supplementary material for: The SNP (rs2230500) in PRKCH Decreases the Risk of Carotid Intima-Media Thickness in a Chinese Young Adult Population
Source: PLoS One. 2012 Jul 11;7(7):e40606. doi: 10.1371/journal.pone.0040606 (PMC3394745; doi:10.1371/journal.pone.0040606)
Supplement: Table S4 — The effect of interaction between genotypes and hypertension status on the atherosclerosis diagnosed by CIMT. (DOC) [file pone.0040606.s004.doc]

**Supporting Information**

**Table S4. The effect of interaction between genotypes and hypertension status on the atherosclerosis diagnosed by CIMT.**

|  |  | Left | |  | Right | |
| --- | --- | --- | --- | --- | --- | --- |
| Hypertension | Recessive model | OR (95%CI)* | *P** |  | OR (95%CI)* | *P** |
| Yes | GG+AG | 1 |  |  | 1 |  |
| Yes | AA | - | - |  | - | - |
| No | GG+AG | 0.43 (0.28-0.67) | <0.001 |  | 0.33 (0.21-0.52) | <0.001 |
| No | AA | 0.47 (0.18-1.26) | 0.135 |  | 0.49 (0.18-1.32) | 0.157 |

*Adjusted for age and gender.
