# Supplementary material for: The SNP (rs2230500) in PRKCH Decreases the Risk of Carotid Intima-Media Thickness in a Chinese Young Adult Population
Source: PLoS One. 2012 Jul 11;7(7):e40606. doi: 10.1371/journal.pone.0040606 (PMC3394745; doi:10.1371/journal.pone.0040606)
Supplement: Method S1 — Allele-specific real-time PCR assay. (DOC) [file pone.0040606.s005.doc]

**Supporting Information**

**Method S1. Allele-specific real-time PCR assay.**

The PCR amplifications were performed using the following primers:

Common primer, 5’-GCAGAATCACGTCCTTCTTCAG-3’;

Allele-specific primer (A), 5’-CATAGGTGATGCTTGCAAGAA-3’;

Allele-specific primer (G), 5’-CATAGGTGATGCTTGCAAGAG-3’.

Individual DNA sample was genotyped for single SNP by using an equal aliquot of samples with 2 allele-specific PCR reactions, each containing 1 of the allele-specific (A-S) primers and a common primer. PCR reaction with the A-S primer that matched the allele in the template DNA amplified normally, whereas PCR reaction with the other A-S primer that mismatched the allele in the template was prevented or delayed when PCR reaction was monitored in real-time (by including SYBR Green I in the PCR and following fluorescence cycle-by-cycle). For each amplification, a fluorescence threshold near the baseline fluorescence was used to calculate a cycle threshold value, which was then used to call the genotype of the sample. PCR was carried out on the GeneAmp 5700 Sequence Detector with procedure of 12 minutes at 95°C, followed by 45 cycles of 30 seconds at 95°C, 30 seconds at 58°C, and finished by 20 minutes dissociation at 60°C. Genotype was directly obtained with the GeneAmp 5700 SDS software.
